# Preserved C-peptide secretion is associated with fewer low-glucose events and lower glucose variability on flash glucose monitoring in adults with type 1 diabetes

**DOI:** 10.1007/s00125-020-05099-3

**Published:** 2020-02-07

**Authors:** Fraser W. Gibb, John A. McKnight, Catriona Clarke, Mark W. J. Strachan

**Affiliations:** 1 Edinburgh Centre for Endocrinology & Diabetes, Royal Infirmary of Edinburgh, Little France Crescent, Edinburgh, EH16 4SA UK; 2 Centre for Cardiovascular Science, University of Edinburgh, Edinburgh, UK; 3 Edinburgh Centre for Endocrinology & Diabetes, Western General Hospital, Edinburgh, UK; 4 Department of Clinical Biochemistry, Western General Hospital, Edinburgh, UK

**Keywords:** Clinical diabetes, Continuous glucose monitoring, C-peptide, Devices, Hypoglycaemia

## Abstract

**Aims/hypothesis:**

We aimed to assess whether persistence of C-peptide secretion is associated with less glucose variability and fewer low-glucose events in adults with type 1 diabetes who use flash monitoring.

**Methods:**

We performed a cross-sectional study of 290 adults attending a university teaching hospital diabetes clinic, with type 1 diabetes, who use flash monitoring and in whom a random plasma C-peptide was available in the past 2 years. Variables relating to flash monitoring were compared between individuals with low C-peptide (<10 pmol/l) and those with persistent C-peptide (either 10–200 pmol/l or 10–50 pmol/l). In addition, the relationship between self-reported hypoglycaemia and C-peptide was assessed (*n* = 167). Data are median (interquartile range).

**Results:**

Individuals with preserved C-peptide secretion (10–200 pmol/l) had shorter duration of diabetes (15 [9–24] vs 25 [15–34] years, *p* < 0.001) and older age at diagnosis (23 [14–28] vs 15 [9–25] years, *p* < 0.001), although current age did not differ in this cohort. Preserved C-peptide was associated with lower time with glucose <3.9 mmol/l (3% [2–6%] vs 5% [3–9%], *p* < 0.001), fewer low-glucose events per 2 week period (7 [4–10] vs 10 [5–16], *p* < 0.001), lower SD of glucose (3.8 [3.4–4.2] vs 4.1 [3.5–4.7] mmol/l, *p* = 0.017) and lower CV of glucose (38.0 [35.0–41.6] vs 41.8 [36.5–45.8], *p* < 0.001). These differences were also present in those with C-peptide 10–50 pmol/l and associations were independent of diabetes duration and estimated HbA_1c_ in logistic regression analysis. Preserved C-peptide was also associated with lower rates of self-reported asymptomatic hypoglycaemia (8.0% vs 22.8% in the past month, *p* = 0.028).

**Conclusions/interpretation:**

Preserved C-peptide secretion is associated with fewer low-glucose events and lower glucose variability on flash monitoring. This suggests that individuals with preserved C-peptide may more safely achieve intensive glycaemic targets.

**Electronic supplementary material:**

The online version of this article (10.1007/s00125-020-05099-3) contains peer-reviewed but unedited supplementary material, which is available to authorised users.

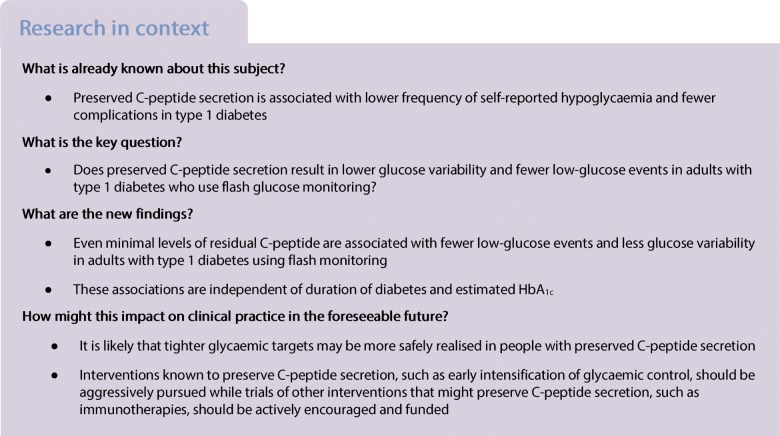



## Introduction

In recent years the utility of C-peptide measurement in routine clinical diabetes practice has garnered increasing attention [1]. C-peptide measurement in those with a clinician diagnosis of type 1 diabetes is an effective means of identifying individuals who may have monogenic diabetes [2] and of identifying other misclassifications (e.g. type 2 diabetes), with the potential for significant changes in therapy. Even in those with a clear diagnosis of type 1 diabetes, there is increasing evidence that confirming endogenous insulin secretion has clinical implications. Significant residual C-peptide secretion is more likely in those diagnosed in adulthood, ranging from 36% within the first 5 years to 22% up to 20 years post diagnosis [3], with evidence of stabilisation after an exponential fall in the first 7 years [4]. Even after 50 years of type 1 diabetes, 32.4% of individuals retain detectable C-peptide levels (>16 pmol/l) [5]. Evidence from the DCCT suggests that intensive therapy may prolong the duration of C-peptide persistence [6]. In the DCCT, preserved C-peptide secretion was associated with lower rates of severe hypoglycaemia [7], fewer diabetes complications, lower HbA_1c_ and lower insulin doses [8], although these associations were mostly observed in the intensive treatment arm. These associations were not noted in the recent Joslin Medalist Study which assessed individuals with long-standing type 1 diabetes [5]. Preservation of C-peptide has been associated with lower self-reported rates of symptomatic, asymptomatic and severe hypoglycaemia, although not with measures of impaired awareness, in a number of observational studies [9–11]. Many C-peptide assays in current clinical use provide a limit of quantification at around 50 pmol/l; however, C-peptide levels >10 pmol/l have been independently associated with a lower risk of diabetes complications [10]. C-peptide persistence has been associated with improved continuous glucose monitoring (CGM) ‘time in range’ in a largely paediatric cohort of people with recently diagnosed type 1 diabetes [12] and with lower glucose variability and low-glucose events in type 2 diabetes [11]. However, little is known of the effects in adults with type 1 diabetes and beyond the first few years after diagnosis. Within the past 2 years, our centre has expanded use of flash glucose monitoring to approximately 50% of individuals with type 1 diabetes [13]. Across a similar timescale we have introduced a programme to measure random plasma C-peptide in all individuals with apparent type 1 diabetes of more than 3 years’ duration. The convergence of these two events has provided the opportunity to assess the relationship between C-peptide status and flash glucose monitoring variables in a large cohort of adults with type 1 diabetes in a ‘real-world’ clinical context. We hypothesised that persistence of C-peptide, even at low levels, would be associated with less glucose variability and fewer low-glucose events.

## Methods

### Study design and participants

We conducted a cross-sectional study of adults with type 1 diabetes, using flash glucose monitoring (Freestyle Libre, Abbott, Witney, UK), in whom random plasma C-peptide results were available. Since July 2017, we have routinely measured random C-peptide in all people with type 1 diabetes of greater than 3 years’ duration in our centre (comprising the Royal Infirmary of Edinburgh and Western General Hospital diabetes clinics), to help identify potential misclassifications (e.g. monogenic diabetes or type 2 diabetes). Our centre approved National Health Service-funded flash glucose monitoring for all individuals meeting Scottish Diabetes Group criteria in February 2018 [13]. To date, approximately 50% of our type 1 diabetes population have commenced flash monitoring. All flash monitoring users are encouraged to link their data to our centre using the LibreView platform (Abbott, Witney, UK). To be included in this study, the following criteria had to be met:

- diabetes duration >3 years;
- concomitant plasma glucose >4 mmol/l at time of C-peptide measurement;
- 2 weeks of flash monitoring data available from LibreView between February and April 2019;
- ≥75% data capture with respect to flash monitoring data within the 2 week period being assessed.

Individuals with C-peptide >200 pmol/l (*n* = 21) were excluded from this analysis to limit the possibility of including people with diagnoses other than type 1 diabetes. All elements of this observational study reflect routine clinical care and therefore ethics approval was not required. These data are presented with the consent of the owner (NHS Lothian).

### Outcomes

The principal analyses compared individuals with C-peptide <10 pmol/l (low) and those with C-peptide 10–200 pmol/l (preserved). A further category was created to compare low C-peptide (<10 pmol/l) with ‘micro-secretors’ (10–50 pmol/l). The main outcomes of interest were differences in flash monitoring variables (obtained from the LibreView platform) between those with low and preserved C-peptide. The key flash monitoring variables were average glucose, SD of glucose, CV of glucose, number of low-glucose events (<3.9 mmol/l) per 2 weeks, time below range (glucose <3.9 mmol/l), time in range (glucose 3.9–10.0 mmol/l), time above range (glucose >10 mmol/l), low-glucose event average duration, estimated HbA_1c_ and interquartile range (IQR) of glucose. Most recent HbA_1c_ measurement, age at diagnosis, diabetes duration, BMI and treatment type (continuous subcutaneous insulin infusion [CSII] or multiple daily injections [MDI]) were obtained from our national clinic database system, SCI-Diabetes (https://www.sci-diabetes.scot.nhs.uk). Presence of microvascular complications was derived from SCI-Diabetes and the electronic patient record. Individuals attending the Royal Infirmary of Edinburgh are routinely asked to complete a hypoglycaemia questionnaire at each clinic visit, which includes a modified Clarke questionnaire [14] and Gold score [15] (available in 167/187 [89.3%]).

### Assays

Random plasma C-peptide was measured using an Abbott Architect immunoassay. In-house studies have demonstrated a CV of 7% at 7 pmol/l and of 15% at 4 pmol/l. Based on these data, we report 4 pmol/l as the limit of quantification in this study. HbA_1c_ was measured by ion-exchange high performance liquid chromatography using the Arkray Adams A1c automated platform (A. Menarini Diagnostics, Winnersh, UK).

### Statistical analysis

Data were mostly non-normally distributed (as determined by Shapiro–Wilk test) and are presented as median and IQR. Unpaired data were analysed by Mann–Whitney *U* test. Categorical data were compared by χ^2^ or by Fisher’s exact test where the conditions for χ^2^ were not met. Correlations were analysed by Spearman’s rank correlation. Logistic regression was performed to identify independent predictors of key flash monitoring variables. Significance was accepted at *p* < 0.05. All analyses were performed using RStudio version 1.0.153 (https://www.rstudio.com).

## Results

### Participant characteristics

**Fig. 1:**
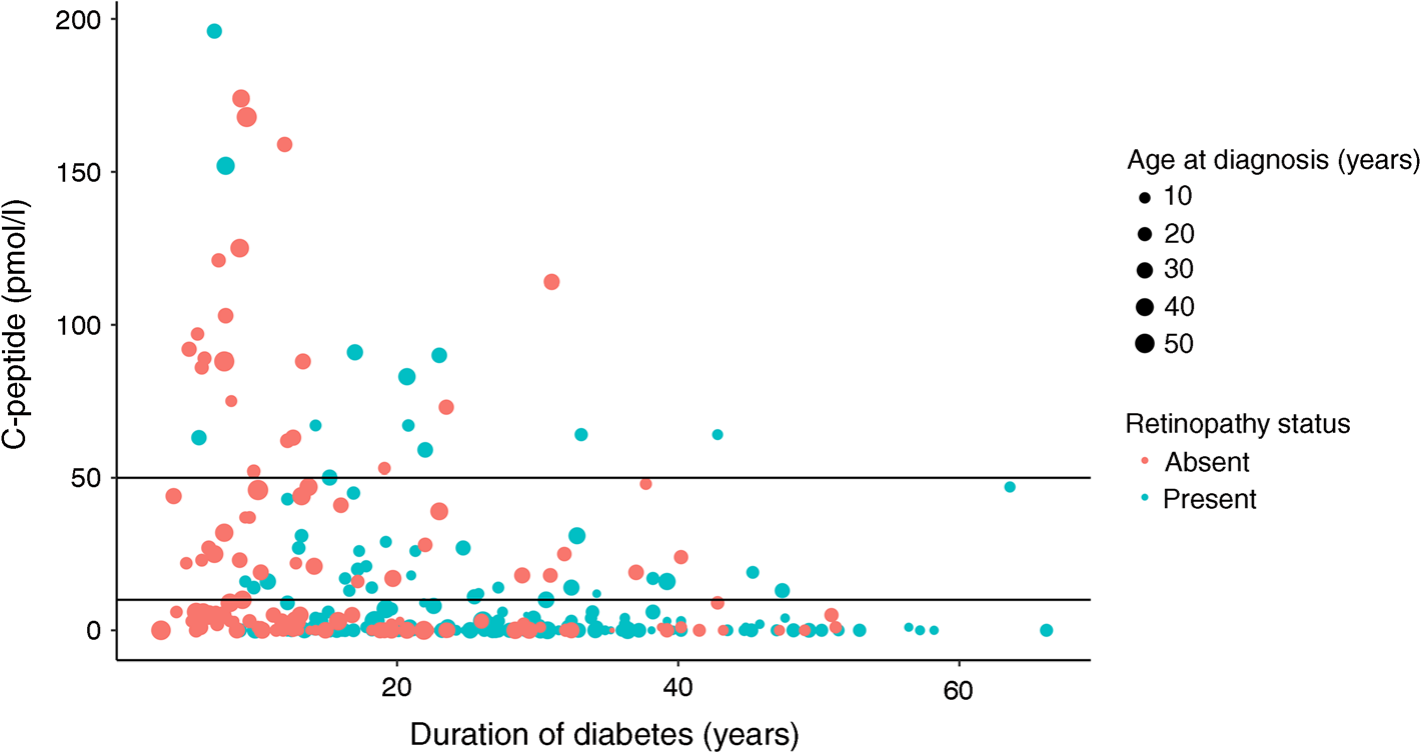
Relationship between diabetes duration and random plasma C-peptide. Blue dots represent individuals with any reported retinopathy and red dots represent those with no retinopathy. Size of dot corresponds to age at diagnosis. Horizontal lines represent 10 pmol/l and 50 pmol/l thresholds

Full characteristics are presented in Table 1, comparing those with low (<10 pmol/l) and preserved (10–200 pmol/l) C-peptide or micro-secretion of C-peptide (10–50 pmol/l). Median C-peptide was <4 pmol/l (<4 to <4) in the low group, 22 pmol/l (16–31) in the micro-secretion group and 32 pmol/l (19–67) in the preserved group. C-peptide concentration was negatively correlated with duration of diabetes (*r* −0.362, *p* < 0.001) (Fig. 1), and although presence of retinopathy (of any severity) was more common in low-C-peptide individuals, this association was not independent of diabetes duration. Neither severe retinopathy (i.e. requiring either retinal photocoagulation or specialist ophthalmology review) nor elevated urinary albumin/creatinine ratio was associated with C-peptide status (Table 1). C-peptide was not significantly correlated with plasma glucose at the time of C-peptide measurement (*r* 0.044, *p* = 0.460) or most recent HbA_1c_ (*r* 0.040, *p* = 0.450). In comparison with the other 2597 individuals with type 1 diabetes attending our centre, participants in this study had lower HbA_1c_ (58 [52–66] vs 63 [54–74] mmol/mol, *p* < 0.001) (7.5% [6.9%–8.2%] vs 7.9% [7.1%–8.9%]), were more likely to use CSII (28.3% vs 13.4%, *p* < 0.001), were younger (42 [31–53] vs 46 [31–59] years, *p* = 0.016) and had longer duration of diabetes (21 [13–32] vs 19 [10–32] years, *p* = 0.007).

**Table 1:** Comparison of clinical and demographic variables by C-peptide status

Variable	<10 pmol/l *n* = 201	10–200 pmol/l *n* = 89	*p* for <10 pmol/l vs 10–200 pmol/l	10–50 pmol/l *n* = 58	*p* for <10 pmol/l vs 10–50 pmol/l
Age at diagnosis (years)	15 (9–25)	23 (14–28)	<0.001	21 (14–30)	<0.001
Duration of diabetes (years)	25 (15–34)	15 (9–24)	<0.001	17 (10–28)	<0.001
Current age (years)	43 (31–53)	39 (31–53)	0.560	41 (31–54)	0.975
BMI (kg/m^2^)	26.6 (23.7–30.0)	27.2 (24–30.6)	0.327	27.2 (24.2–30.7)	0.319
HbA_1c_ (mmol/mol)	57 (52–67)	58 (52–65)	0.845	57 (52–64)	0.607
HbA_1c_ (%)	7.4 (6.9–8.3)	7.5 (6.9–8.1)		7.4 (6.9–8.0)	
Obese	51/200 (25.5%)	27/89 (30.3%)	0.392	19/58 (32.8%)	0.274
Male	108/201 (53.7%)	53/89 (59.6%)	0.358	34/58 (58.6%)	0.510
CSII	60/201 (29.9%)	22/89 (24.7%)	0.371	16/58 (27.6%)	0.739
Any retinopathy	137/201 (68.2%)	42/89 (47.2%)	<0.001	27/58 (46.6%)	0.039
Any retinal photocoagulation therapy	35/201 (17.4%)	11/89 (12.4%)	0.277	10/58 (17.2%)	0.976
Under specialist ophthalmology review	53/201 (26.4%)	17/89 (19.1%)	0.182	14/58 (24.1%)	0.733
Elevated urinary albumin/creatinine ratio	29/200 (14.5%)	14/88 (15.9%)	0.757	10/58 (17.2%)	0.608

Data are presented as median (IQR) or as *n*/*N* (%)

### Flash glucose data

**Fig. 2:**
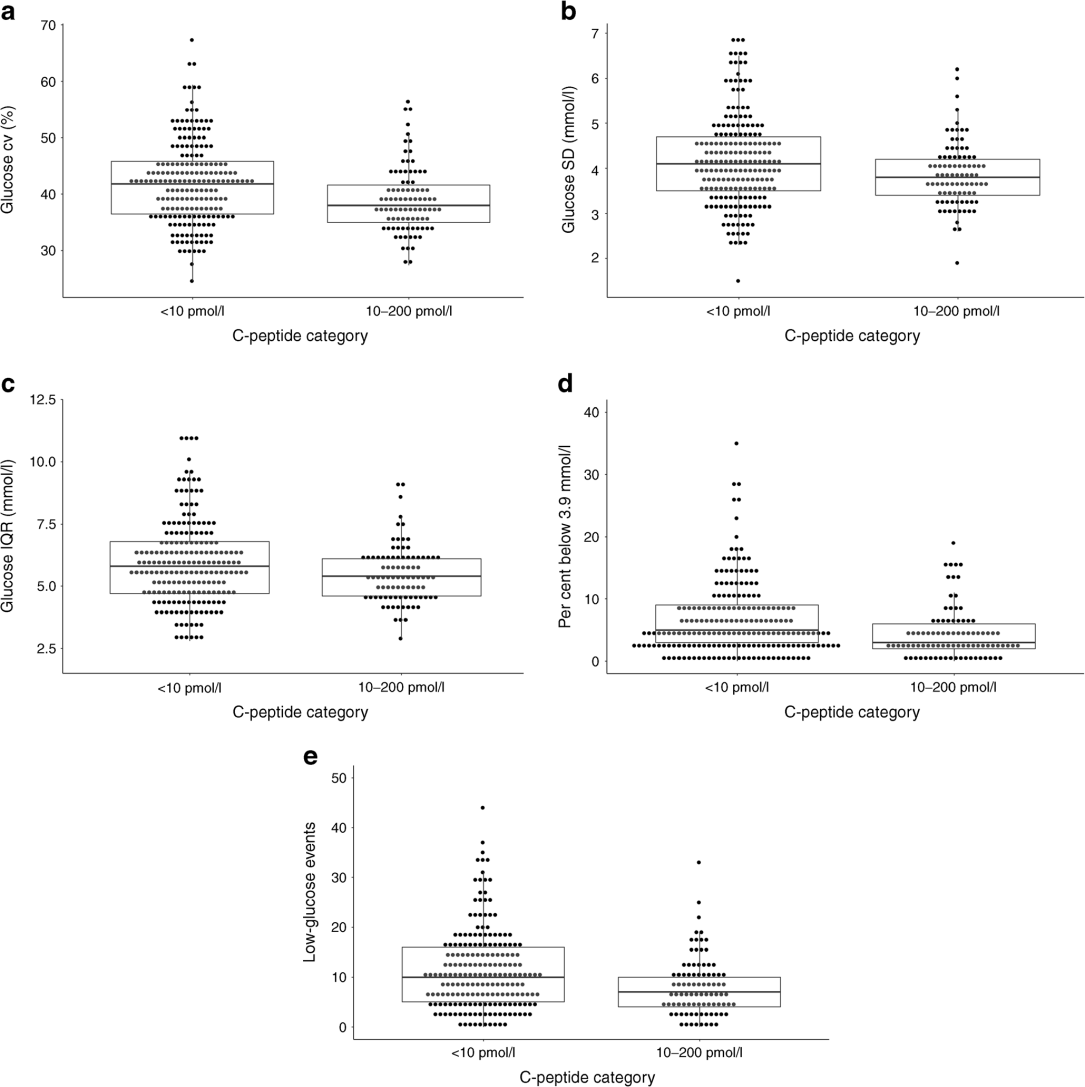
Influence of C-peptide category upon the following flash glucose data: (**a**) CV, (**b**) SD, (**c**) IQR, (**d**) percentage of time below 3.9 mmol/l and (**e**) low-glucose events per 14 days. The boxes represent median and IQR and whiskers represent 1.5 × IQR

Average glucose, estimated HbA_1c_, time in range and time above range did not differ significantly between those with C-peptide <10 pmol/l and those with preserved C-peptide secretion (Table 2). However, CV, SD and IQR were all lower in individuals with preserved C-peptide (Table 2 and Fig. 2). Similarly, time below range and number of low-glucose events were lower in individuals with preserved C-peptide (Table 2 and Fig. 2). When limiting the comparison to those with C-peptide levels of 10–50 pmol/l, these associations remained significantly different compared with low-C-peptide individuals (Table 2). C-peptide was significantly correlated with CV (*r* −0.141, *p* = 0.016), time below range (*r* −0.180, *p* = 0.002) and low-glucose events (*r* −0.182, *p* = 0.002) (electronic supplementary material [ESM] Fig. 1a–c).

**Table 2:** Comparison of data derived from flash monitoring by C-peptide category

Variable	<10 pmol/l *n* = 201	10–200 pmol/l *n* = 89	*p* for <10 pmol/l vs 10–200 pmol/l	10–50 pmol/l *n* = 58	*p* for <10 pmol/l vs 10–50 pmol/l
Average glucose (mmol/l)	9.8 (8.7–11.0)	9.8 (8.9–11.0)	0.792	9.5 (8.8–10.4)	0.470
SD (mmol/l)	4.1 (3.5–4.7)	3.8 (3.4–4.2)	0.017	3.8 (3.4–4.2)	0.032
CV (%)	41.8 (36.5–45.8)	38.0 (35.0–41.6)	<0.001	38.5 (35.1–44.4)	0.030
Low events per 2 weeks (*n*)	10 (5–16)	7 (4–10)	<0.001	8.0 (4.3–12.8)	0.037
Below 3.9 mmol/l (%)	5 (3–9)	3 (2–6)	<0.001	4 (2–6)	0.034
In range (3.9–10.0 mmol/l) (%)	50 (39–58)	52 (42–61)	0.448	53 (45–62)	0.117
Above 10 mmol/l (%)	44 (32–55)	45 (34–55)	0.637	42 (33–50)	0.565
Low event average duration (min)	100 (76–127)	90 (66–120)	0.138	90 (66–123)	0.302
Estimated HbA_1c_ (mmol/mol)	62 (54–69)	62 (55–69)	0.794	59 (55–66)	0.464
Estimated HbA_1c_ (%)	7.8 (7.1–8.5)	7.8 (7.2–8.5)		7.6 (7.2–8.2)	
IQR (mmol/l)	5.8 (4.7–6.8)	5.4 (4.6–6.1)	0.028	5.3 (4.6–6.0)	0.032

Data are presented as median (IQR)

Logistic regression models identified C-peptide status as independently contributing to associations with glucose variability and low-glucose variables (Table 3).

**Table 3:** Logistic regression analysis results assessing factors associated with a flash monitoring data result above the median for this cohort

Variable	SD above median (4.0 mmol/l)		CV glucose above median (40.3%)		IQR glucose above median (5.6 mmol/l)		Low events per 2 weeks above median (nine events)		Percentage of time below 3.9 mmol/l above median (4%)	
	OR (95% CI)	*p*	OR (95% CI)	*p*	OR (95% CI)	*p*	OR (95% CI)	*p*	OR (95% CI)	*p*
C-peptide 10–200 pmol/l	0.32 (0.16–0.63)	0.001	0.30 (0.17–0.52)	<0.001	0.40 (0.21–0.75)	0.005	0.29 (0.15–0.54)	<0.001	0.32 (0.17–0.60)	<0.001
Duration of diabetes (per year)	1.00 (0.98–1.03)	0.841	0.99 (0.96–1.00)	0.194	1.00 (0.98–1.03)	0.776	0.97 (0.95–1.00)	0.021	0.98 (0.96–1.00)	0.074
Estimated HbA_1c_ (per mmol/mol)	1.16 (1.12–1.21)	<0.001	0.98 (0.96–1.00)	0.019	1.37 (1.10–1.18)	<0.001	0.89 (0.86–0.91)	<0.001	0.88 (0.84–0.91)	<0.001
Male	1.34 (0.72–2.50)	0.355	1.70 (1.03–2.84)	0.040	1.32 (0.73–2.38)	0.359	1.18 (0.67–2.10)	0.567	1.19 (0.67–2.15)	0.552
CSII	0.82 (0.42–1.61)	0.573	1.00 (0.57–1.77)	0.976	0.94 (0.50–1.80)	0.862	0.68 (0.36–1.29)	0.245	1.05 (0.55–2.00)	0.888

### Hypoglycaemia questionnaire data

Median Gold score (2 [1–2] in low vs 1 [1–2] in preserved, *p* = 0.506) and the percentage of people with impaired awareness of hypoglycaemia, defined as Gold score ≥4 (9.6% vs 2.4%, *p* = 0.281), did not significantly differ by C-peptide status. Individuals with more than 2–3 episodes of symptomatic hypoglycaemia per week, in the preceding month, did not differ significantly between groups (53.5% vs 40.8%, *p* = 0.137). However, low-C-peptide individuals were more likely to report any asymptomatic hypoglycaemia in the past month (22.8% vs 8.0%, *p* = 0.028) and to report not always being aware of hypoglycaemia (45.6% vs 28.0%, *p* = 0.034).

## Discussion

As hypothesised, we demonstrated significant associations between residual C-peptide secretion and lower glucose variability and low-glucose events in flash glucose monitoring users. These associations were independent of prevailing HbA_1c_ and diabetes duration, suggesting routine evaluation of C-peptide may have clinical utility in the management of type 1 diabetes. These data also highlight the limitations of HbA_1c_, which did not differ between groups, as a means of assessing optimal glycaemic management. In the modern era, it is conceivable that HbA_1c_ will be supplanted by CGM metrics to optimise glycaemic management [16], particularly in efforts to minimise glucose variability, which has been posited as an independent risk factor for diabetes complications [17].

Our flash glucose monitoring findings are accompanied by significant differences in self-reported hypoglycaemia, consistent with findings from several previous investigations [9–11], although significant differences were limited to asymptomatic hypoglycaemia in our study. Marren et al [9] describe significant differences in self-reported symptomatic and asymptomatic hypoglycaemia in those with preserved C-peptide, although the median C-peptide concentration in this group (114 pmol/l) was substantially higher than in our study (32 pmol/l). While we report random plasma C-peptide and Marren et al [9] used values after a standard mixed-meal tolerance test, the correlation between random and post-mixed-meal C-peptide is known to be very strong (*R* = 0.91) and is unlikely to substantially limit the comparability of these studies [18]. Other important contrasts with this study are the higher HbA_1c_ (67–69 mmol/mol [8.3–8.5%] vs 57–58 mmol/mol [7.4–7.5%] in our cohort) and significant difference in age between C-peptide groups, which was not different in our study (which was limited to adults) despite differences in diabetes duration and age at diagnosis. Kuhtreiber et al [10] demonstrated similar associations between hypoglycaemia and fasting C-peptide, although the median concentration of C-peptide associated with mild and moderate hypoglycaemia (42.4 pmol/l) was higher than the median in both our preserved and micro-secretor categories. The novelty of our self-reported hypoglycaemia data is the demonstration of lower rates of asymptomatic hypoglycaemia at an even lower C-peptide threshold than previously demonstrated [9–11].

The fact that C-peptide status is strongly associated with reduced low-glucose events and glucose variability, but not average glucose, time in range or time above range, offers a mechanistic insight into the consequences of preserved C-peptide. If this were simply a ‘buffering’ effect of ongoing endogenous insulin secretion, smoothing out the peaks and troughs in glucose in individuals receiving exogenous insulin, we might expect to see this reflected in a lower HbA_1c_. However, this was not the case in either our cohort or that of Marren et al [9]. Loss of the glucagon counter-regulatory response to hypoglycaemia occurs in many individuals with type 1 diabetes within 5 years of diagnosis and is linked, in part, to the loss of a paracrine effect of endogenous insulin on alpha cells in pancreatic islets [19]. Adults with type 1 diabetes and preserved C-peptide (>99 pmol/l after a mixed-meal tolerance test) have relative preservation of the glucagon response to hypoglycaemia [20]. Therefore, the role of intra-islet insulin signalling offers a compelling mechanism to explain the association of preserved C-peptide with reduced low-glucose events [21, 22], although this phenomenon was not observed in young individuals within 1 year of diagnosis [23]. The only cohorts where preserved C-peptide was associated with lower HbA_1c_ were the intensive arm of DCCT [8] and the study by Kuhtreiber et al [10], both of which had comparatively low HbA_1c_ levels. Cohorts with no difference in HbA_1c_, in relation to C-peptide status (including this study), may reflect a failure to intensify glycaemic management in individuals who would be at lower risk of hypoglycaemia [9].

The key strength of this study is its novelty in assessing the relationship between C-peptide and flash glucose monitoring variables in a ‘real-world’ clinical context. Where associations of CGM with C-peptide were reported previously, this was in a largely paediatric population, within 2 years of diagnosis and with relatively higher levels of C-peptide [12]. Our cohort also benefits from being balanced in terms of current age and HbA_1c_ between low and preserved C-peptide groups. As a ‘real-world’ assessment, the various measures obtained in this study (questionnaire data, HbA_1c_, C-peptide and flash monitoring data) were not captured simultaneously, although we would envisage this increasing the likelihood of a type II error rather than producing false positive associations with C-peptide. Random C-peptide appears to be as robust a measure of C-peptide status as values obtained after a mixed-meal tolerance test [18], and indeed we found no significant correlation between C-peptide and concomitant plasma glucose. It would have been preferable to have access to different low-glucose thresholds (e.g. <3 mmol/l); however, the nature of the data capture process did not permit this. It would also have been useful to have reported insulin dose data, but unfortunately these were not consistently available. The decision to exclude individuals with C-peptide >200 pmol/l was pragmatic, to limit the likelihood of including misclassified cases, but also because the specific research question related to the effect of relatively low levels of C-peptide secretion. We did not measure diabetes antibodies as a matter of routine and so it is possible that our cohort contained a very small proportion of individuals with a cause of insulin deficiency other than type 1 diabetes, e.g. hepatocyte nuclear factor 1-β monogenic diabetes. However, this does not affect the central tenet of our study regarding the relationship between C-peptide and low-glucose events. While the ‘real-world’ design is a strength in terms of generalisability, our cohort is skewed towards people with lower HbA_1c_ and greater CSII usage than our centre’s total type 1 population.

These findings suggest that routine clinical measurement of C-peptide in type 1 diabetes may be important not only in confirming the correct diagnosis of diabetes, but also in informing the risk of low glucose and glycaemic instability. Given that we have shown an effect in the C-peptide range 10–50 pmol/l, we suggest that wider availability of higher-sensitivity C-peptide assays may be of value, as many currently available clinical assays report 50 pmol/l as their lower limit of quantification. Our findings support previous conclusions, drawn from self-reported hypoglycaemia [9], that there appears to be a failure of intensification in glucose-lowering therapy in people who are at lower risk of hypoglycaemia and glucose variability. These data also raise the possibility of stratified glycaemic targets which acknowledge the influence of residual C-peptide secretion. Given the apparent importance of persistent C-peptide secretion, every effort should be made to ensure early intensification of glycaemic control at diagnosis, as this is currently the only available intervention shown to preserve C-peptide secretion [6]. Moreover, studies of novel strategies to preserve C-peptide secretion, such as immunotherapies, should be supported and funded [24].

## Electronic supplementary material

ESM Figure 1 (PDF 116 kb)

## Data Availability

The datasets generated during and/or analysed during the current study are available from the corresponding author on reasonable request.

## Abbreviations

CGM: Continuous glucose monitoring
CSII: Continuous subcutaneous insulin infusion
IQR: Interquartile range

## References

[1] Jones AG, Hattersley AT (2013). The clinical utility of C-peptide measurement in the care of patients with diabetes. Diabet Med.

[2] Shields BM, Shepherd M, Hudson M (2017). Population-based assessment of a biomarker-based screening pathway to aid diagnosis of monogenic diabetes in young-onset patients. Diabetes Care.

[3] Davis AK, DuBose SN, Haller MJ (2015). Prevalence of detectable C-peptide according to age at diagnosis and duration of type 1 diabetes. Diabetes Care.

[4] Shields BM, McDonald TJ, Oram R (2018). C-peptide decline in type 1 diabetes has two phases: an initial exponential fall and a subsequent stable phase. Diabetes Care.

[5] Yu MG, Keenan HA, Shah HS (2019). Residual β cell function and monogenic variants in long-duration type 1 diabetes patients. J Clin Invest.

[6] The Diabetes Control and Complications Trial Research Group (1998). Effect of intensive therapy on residual beta-cell function in patients with type 1 diabetes in the diabetes control and complications trial. A randomized, controlled trial. Ann Intern Med.

[7] Steffes MW, Sibley S, Jackson M, Thomas W (2003). Beta-cell function and the development of diabetes-related complications in the diabetes control and complications trial. Diabetes Care.

[8] Lachin JM, McGee P, Palmer JP, DCCT/EDIC Research Group (2014). Impact of C-peptide preservation on metabolic and clinical outcomes in the Diabetes Control and Complications Trial. Diabetes.

[9] Marren SM, Hammersley S, McDonald TJ (2019). Persistent C-peptide is associated with reduced hypoglycaemia but not HbA_1c_ in adults with longstanding type 1 diabetes: evidence for lack of intensive treatment in UK clinical practice?. Diabet Med.

[10] Kuhtreiber WM, Washer SLL, Hsu E (2015). Low levels of C-peptide have clinical significance for established type 1 diabetes. Diabet Med.

[11] Hope SV, Knight BA, Shields BM (2018). Random non-fasting C-peptide testing can identify patients with insulin-treated type 2 diabetes at high risk of hypoglycaemia. Diabetologia.

[12] Buckingham B, Cheng P, Beck RW (2015). CGM-measured glucose values have a strong correlation with C-peptide, HbA_1c_ and IDAAC, but do poorly in predicting C-peptide levels in the two years following onset of diabetes. Diabetologia.

[13] Tyndall V, Stimson RH, Zammitt NN (2019). Marked improvement in HbA_1c_ following commencement of flash glucose monitoring in people with type 1 diabetes. Diabetologia.

[14] Clarke WL, Cox DJ, Gonder-Frederick LA, Julian D, Schlundt D, Polonsky W (1995). Reduced awareness of hypoglycemia in adults with IDDM. A prospective study of hypoglycemic frequency and associated symptoms. Diabetes Care.

[15] Gold AE, MacLeod KM, Frier BM (1994). Frequency of severe hypoglycemia in patients with type I diabetes with impaired awareness of hypoglycemia. Diabetes Care.

[16] Battelino T, Danne T, Bergenstal RM (2019). Clinical targets for continuous glucose monitoring data interpretation: recommendations from the international consensus on time in range. Diabetes Care.

[17] Hirsch IB (2015). Glycemic variability and diabetes complications: does it matter? Of course it does!. Diabetes Care.

[18] Hope SV, Knight BA, Shields BM, Hattersley AT, McDonald TJ, Jones AG (2016). Random non-fasting C-peptide: bringing robust assessment of endogenous insulin secretion to the clinic. Diabet Med.

[19] McCrimmon RJ, Sherwin RS (2010). Hypoglycemia in type 1 diabetes. Diabetes.

[20] Fukuda M, Tanaka A, Tahara Y (1988). Correlation between minimal secretory capacity of pancreatic beta-cells and stability of diabetic control. Diabetes.

[21] Gosmanov NR, Szoke E, Israelian Z (2005). Role of the decrement in intraislet insulin for the glucagon response to hypoglycemia in humans. Diabetes Care.

[22] Raju B, Cryer PE (2005). Loss of the decrement in intraislet insulin plausibly explains loss of the glucagon response to hypoglycemia in insulin-deficient diabetes: documentation of the intraislet insulin hypothesis in humans. Diabetes.

[23] Sherr J, Xing D, Ruedy KJ (2013). Lack of association between residual insulin production and glucagon response to hypoglycemia in youth with short duration of type 1 diabetes. Diabetes Care.

[24] Dayan CM, Korah M, Tatovic D, Bundy BN, Herold KC (2019). Changing the landscape for type 1 diabetes: the first step to prevention. Lancet.

