# Supplementary material for: Preserved C-peptide secretion is associated with fewer low-glucose events and lower glucose variability on flash glucose monitoring in adults with type 1 diabetes
Source: Diabetologia. 2020 Feb 7;63(5):906–14. doi: 10.1007/s00125-020-05099-3 (PMC7145780; doi:10.1007/s00125-020-05099-3)

ESM figure 1a: Correlation between CV glucose and C-peptide

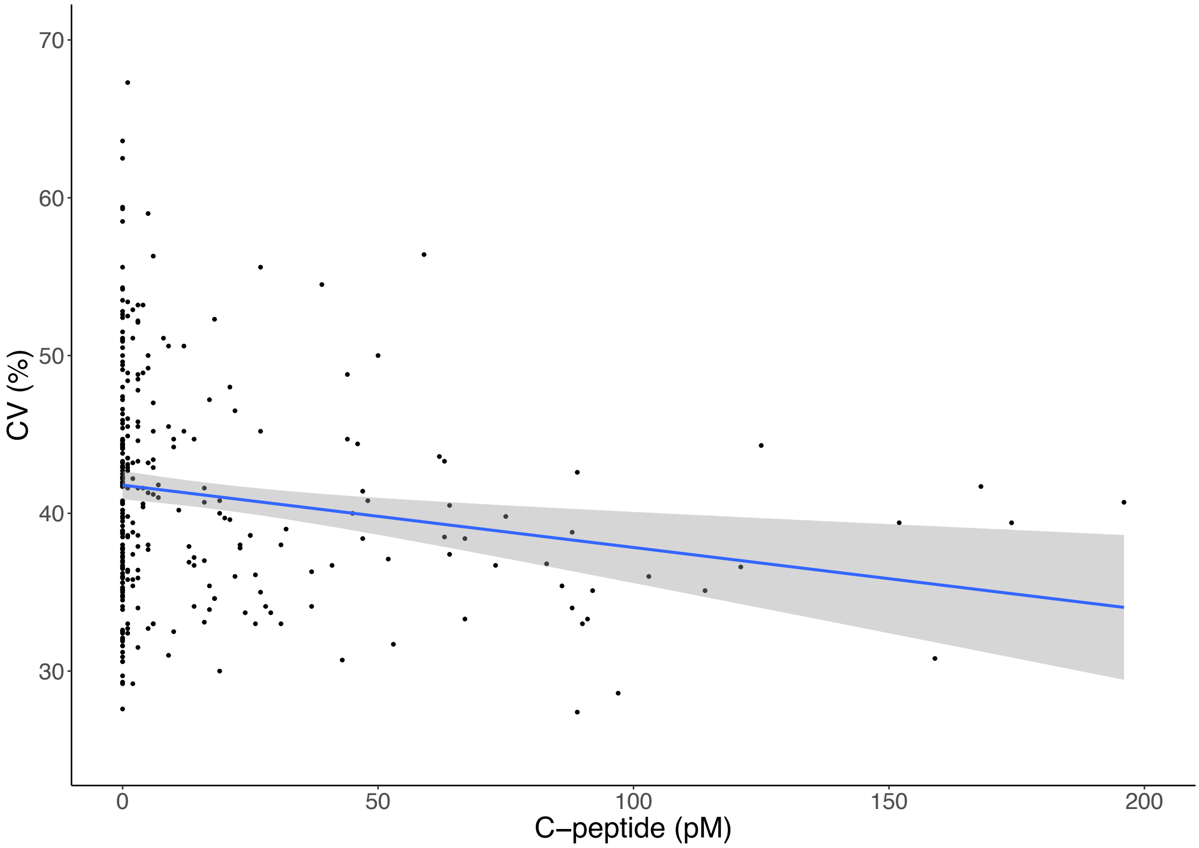

ESM figure 1b: Correlation between % below 3.9mM and C-peptide

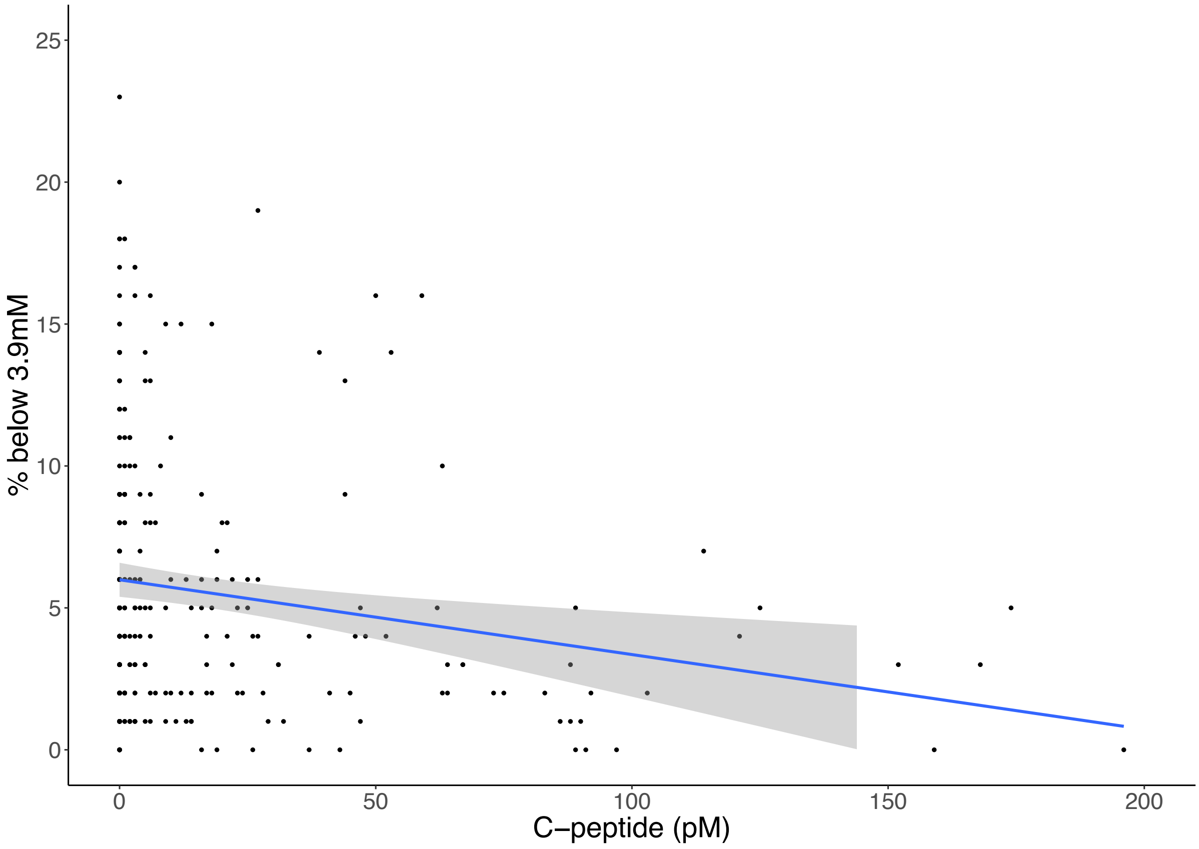

ESM figure 1c: Correlation between low glucose events and C-peptide

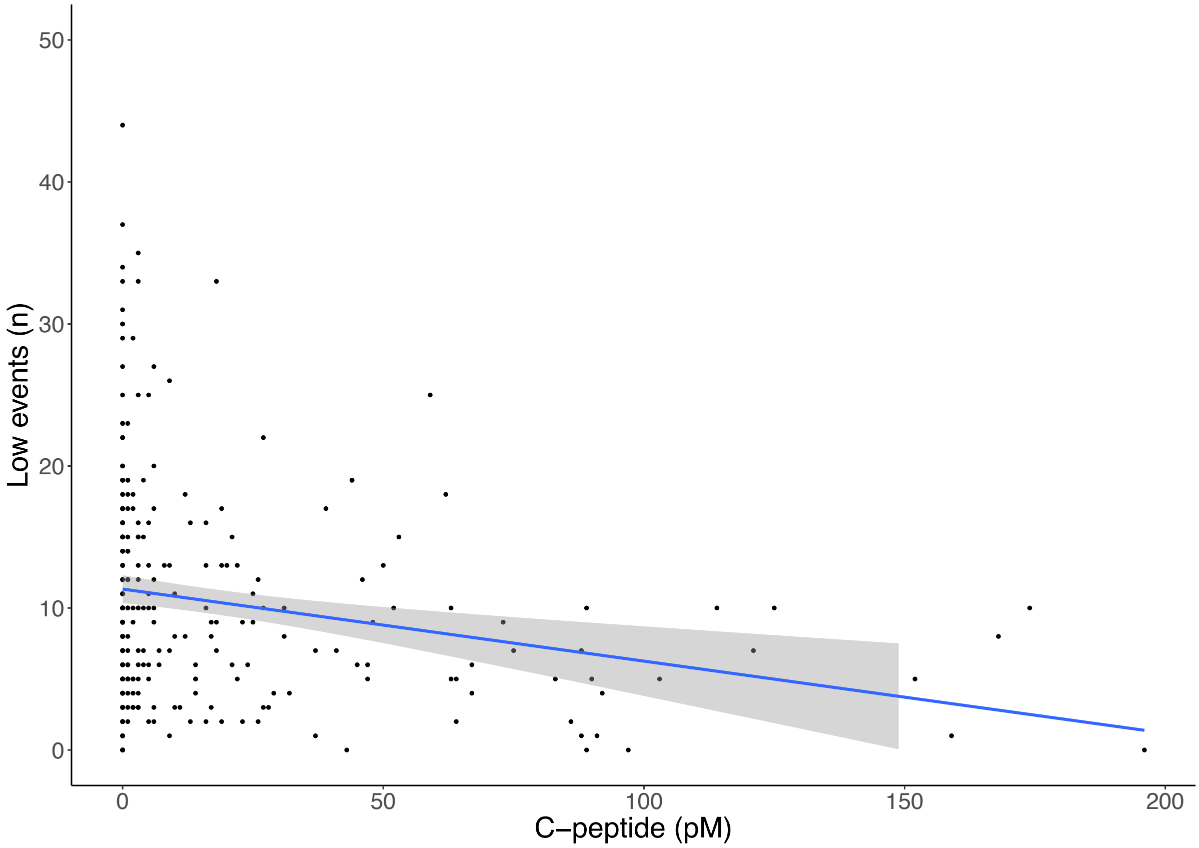

Supplement: Supplementary file 1 — (PDF 116 kb) [file 125_2020_5099_MOESM1_ESM.pdf]
